# Impact of simulation and reference catalogues on the evaluation of taxonomic profiling pipelines

**DOI:** 10.1099/mgen.0.001330

**Published:** 2025-01-13

**Authors:** Vadim Puller, Florian Plaza Oñate, Edi Prifti, Raynald de Lahondès

**Affiliations:** 1 GMT Science 75 route de Lyons-La-Foret, Rouen F-76000, France; 2 IRD, Sorbonne Université, Unité de Modélisation Mathématique et Informatique des Systèmes Complexes, UMMISCO, 32 Avenue Henri Varagnat, Bondy F-93143, France; 3 Sorbonne Université, INSERM, Nutrition et Obesities; Systemic Approaches, NutriOmique, AP-HP, Hôpital Pitié-Salpêtrière, 91 Boulevard de l’Hôpital, Paris F-75013, France

**Keywords:** bias, gut microbiome, profiling tool, reference catalogues, simulation

## Abstract

Microbiome profiling tools rely on reference catalogues, which significantly affect their performance. Comparing them is, however, challenging, mainly due to differences in their native catalogues. In this study, we present a novel standardized benchmarking framework that makes such comparisons more accurate. We decided not to customize databases but to translate results to a common reference to use the tools with their native environment. Specifically, we conducted two realistic simulations of gut microbiome samples, each based on a specific taxonomic profiler, and used two different taxonomic references to project their results, namely the Genome Taxonomy Database and the Unified Human Gastrointestinal Genome. To demonstrate the importance of using such a framework, we evaluated four established profilers as well as the impact of the simulations and that of the common taxonomic references on the perceived performance of these profilers. Finally, we provide guidelines to enhance future profiler comparisons for human microbiome ecosystems: (i) use or create realistic simulations tailored to your biological context (BC), (ii) identify a common feature space suited to your BC and independent of the catalogues used by the profilers and (iii) apply a comprehensive set of metrics covering accuracy (sensitivity/precision), overall representativity (richness/Shannon) and quantification (UniFrac and/or Aitchison distance).

**Impact Statement**

The microbiome field is now reaching a turning point in medical usage, notably with the recent approval of several therapies. However, the question of the accuracy of the microbiome analysis tools, notably the most advanced ones, and the veracity of the inferred observations remains a subject of controversy.

There is a need for robust methods that evaluate the adequacy of microbiome profiling tools for different applications. Typically, most microbiome profiling tools undergo initial benchmarking with previous existing ones, employing several performance metrics quantified on simulated or real metagenomic data. However, these profiling tools rely on distinct reference catalogues, which makes their comparison and performance assessments challenging and biased.

In this article, we introduce a novel computational framework designed to perform a fairer comparison while reducing such biases. This framework employs a comprehensive multi-metric approach that ranks four different tools, MetPhlAn3, MetaPhlAn4, mOTUs3 and Kraken/Bracken. The results reveal that both the simulation methodology and the choice of reference databases significantly influence the evaluation of taxonomic profiling tools.

Finally, our framework provides numerous resources, including a substantial repository of simulated data, code implementations and various software tools, which simplify extensive computations, and we have added a practical guide built on our proposed guidelines (in the code repository provided with the article).

## Data Summary

Supplementary information accompanies this paper as a separate PDF file (see supplementary material). The simulated samples are published on the NCBI SRA archive under the accession PRJNA987980. Code and other data are provided with extensive documentation in https://github.com/gmtsciencedev/microbiome-pipeline-benchmarking.

## Introduction

Over the past decade, the field of microbiome research has grown substantially. A series of landmark publications have firmly established connections between the microbial ecosystem and numerous human diseases. Notably, the gut microbiome has gained prominence as a critical organ and as a sentinel for human diseases [1,9]. Furthermore, research has explored the predictive potential of the gut microbiome, not only for disease diagnosis but also for assessing disease severity [10,12].

Characterizing microbiome samples primarily involves identifying the various species and strains present, along with quantifying their abundance as well as their functional capabilities. Numerous methodologies have been devised to address this challenge. They notably diverge in their sequencing strategies: some are designed to target highly conserved genomic regions, such as the 16S rRNA genes, while others employ a broader sequencing approach known as whole metagenomic sequencing (WMS). Each of these approaches imposes distinct analytical requirements. This study focuses on the analytical methods tailored for WMS data.

The manipulation of WMS data presents several complex challenges owing to their inherent properties such as compositionality and variable interdependence. Consequently, a multitude of WMS taxonomic profilers are available to address these complexities, each offering distinct advantages and limitations. Irrespective of the specific method employed, taxonomic profilers designed for WMS data have a common reliance on reference catalogues. We prefer the term of catalogue to describe this loosely structured collection of reference genomic items, generally genes or complete genomes, which is different from a more structured database. However, some authors use the term database to describe a similar concept. These catalogues have evolved in terms of methodology, size and quality over the past decade. To illustrate, the initial gene reference catalogue for the human gut microbiome was introduced in 2010 [13] and has since expanded exponentially from a mere couple of million genes to tens of millions [14] and eventually to hundreds of millions of genes [15]. The diversity in catalogues across the literature highlights the dynamism of the field, yet it also presents challenges in comparing results across different studies.

Some profilers assign sequenced reads to representative genome catalogues such as Kraken [16,18], Centrifuge [19], Kaiju [20] or DIAMOND [21]. These pipelines may use different catalogues, notably based on RefSeq [22,23] or Genome Taxonomy Database (GTDB) [24,27] or MGnify [28] (see Tables S1 and S2, available in the online Supplementary Material, for a more complete summary of the popular metagenomic profilers and recent benchmarking studies). GTDB has a specific status; it uses genomes from the National Center for Biotechnology Information (NCBI), with specific taxonomic annotations, but it makes a specific selection on reference genomes for each taxonomic entry and can be considered for this reason as a distinct catalogue from the NCBI.

Another class of taxonomic profilers relies on tailor-made catalogues of marker genes such as MetaPhlAn [29,33] or mOTUs [34,35]. These tools benefit from their selection of marker genes and associated taxonomy. However, they may underperform in specific scenarios, such as simulations involving species poorly or not represented in their catalogues [36,37].

The non-redundant gene catalogue-based profilers constitute a third class. They include, for instance, Meteor [38,39], Mocat [40,41] and NGLess [42]. They are like marker gene catalogue profilers but may take advantage of non-marker genes to provide a more comprehensive description. Unfortunately, no recent complete open implementation of this class is available at the time of the writing, so they were not included in this benchmark.

It is worth emphasizing that the most commonly used metrics for assessing species presence (e.g. those derived from confusion matrices [37]) and metrics for comparing abundance profiles (e.g. various distance measures [37]) assume that the compared tools operate within the same ‘feature’ space. In this context, we use the machine learning term, ‘feature’, to denote the various taxonomic units employed by different profilers to describe sample composition. More specifically, mOTUs refer to those as operational taxonomic units (OTUs) and MetaPhlAn as species genome bins, while Kraken refers to them as clades, all of which are assimilated to the species corresponding to the taxonomic annotation in most publications.

Novel microbiome profiling pipelines are systematically compared with existing ones using either mock communities or simulated samples by tools such as CAMISIM [43] or different reference datasets not necessarily focused on the human gut microbiome [44]. Simulations remain inherently reliant on the input genomes and specified abundances. The realism of such simulations further hinges on several factors including the number of distinct species (richness of the ecosystem), the specific composition in terms of biome specialization, the genetic distance between species and the distribution of their relative abundance.

When comparing profilers, the diversity of reference catalogues also poses a specific challenge. Three distinct approaches emerge. The first involves changing default pipeline catalogues to a common one. An alternative approach conducts comparisons at different taxonomic levels (e.g. species, genus or phylum), where disparities between catalogues tend to be less pronounced [45,48]. In the third approach, pipelines use their native catalogues but project their results onto a shared feature space.

In this article, we introduce a novel standardized benchmarking approach that uses shared feature space projections. Our objective was to take advantage of the capabilities of simulation, while maintaining a close semblance to actual gut microbial samples obtained from colorectal cancer patients and control subjects (*n*=343) [11,12]. To achieve this, we applied two distinct profilers on sequenced data from real samples and generated realistic community descriptions for downstream simulations. These two pipelines are representative of a specific class of profilers: Kraken for the genome catalogue-based pipelines and MetaPhlAn4 for the marker gene catalogue-based pipelines. These real samples served as reference abundance profiles for two distinct simulation scenarios. The simulated samples were then analysed using a panel of four different profilers, including the two used for the simulation, to estimate the distribution of taxonomic profiles and the impact of feature spaces. These results were then compared with the reference abundance profiles employed during the simulations. Our evaluation of these tools encompassed a range of standard performance metrics, including the assessments of alpha and beta diversity disparities, as well as precision and sensitivity analyses.

## Methods

### State-of-the-art taxonomic profilers

The taxonomic profilers and classifiers evaluated in this study are Kraken [16,17] [used together with Bayesian Reestimation of Abundance with KrakEN (Bracken)] [18,49], MetaPhlAn3 [29,31], mOTUs3 [34,35] and MetaPhlAn4 [32,33]. These tools were selected based on specific criteria, including the type of method, their popularity in the scientific community measured by a yearly averaged number of citations and how well they are maintained. Below is a short description of each profiler.

### Kraken and Bracken

Kraken uses K-mers to match metagenomic reads to whole genomes. We applied it with the GTDB release R207 [24,27] downloaded from the webpage of Struo2 tool (https://github.com/leylabmpi/Struo2) [50,51]. Kraken was also used with the Unified Human Gastrointestinal Genome (UHGG) collection available on MGnify [28,52], downloaded from the official repository (http://ftp.ebi.ac.uk/pub/databases/metagenomics/mgnify_genomes/human-gut/). As Kraken alone does not determine the species abundance, it is often used together with its sister tool called Bracken [18,49]. Subsequently, we refer to the pipeline composed of Kraken and Bracken simply as *Kraken*. When Kraken was used alone (without Bracken), which was only the case to project native feature spaces to GTDB or UHGG feature spaces (see below), it is referred as *Kraken (alone*).

### MetaPhlAn and mOTUs

MetaPhlAn and mOTUs are two metagenomic profilers relying on marker genes. Both tools come with their own custom marker gene catalogues. Importantly, MetaPhlAn uses species-specific marker genes (e.g. genes shared by all strains of a given species and not found in the strains of other species), whereas mOTUs use a collection of universal single-copy marker genes corresponding to orthologous gene families present in all prokaryotic species. The version of mOTUs used here is mOTUs 3.0.3. MetaPhlAn3 is a popular version of the tool, which has been superseded by the newest MetaPhlAn4 version. MetaPhlAn4 was released when the current study was in preparation, drastically increasing the reference catalogue size by including metagenome-assembled genomes in addition to the cultured species already included in MetaPhlAn3. Therefore, we chose to keep both MetaPhlAn versions in our comparison.

### Experimental workflow

**Fig. 1.**
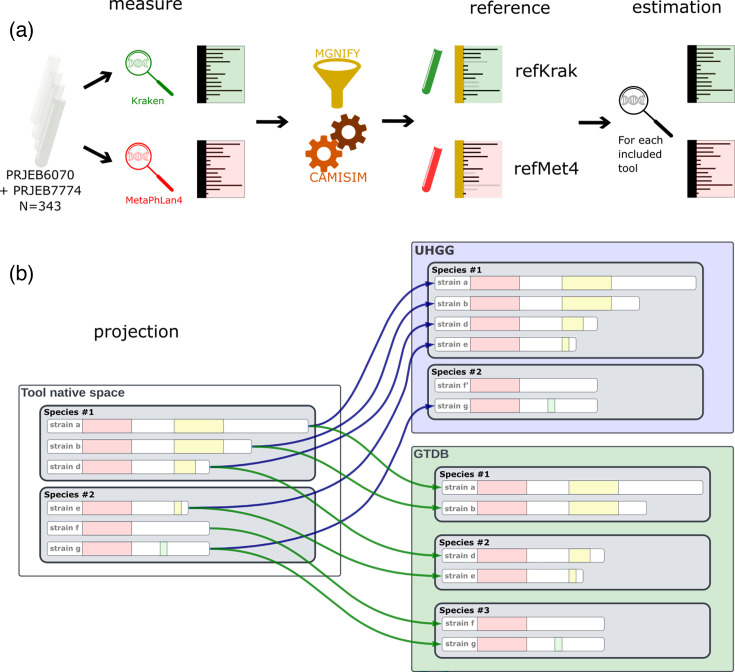
Schematic representation of the simulation and the analyses carried out in this study. (**a**) Samples from the PRJEB6070 and PRJEB7774 bioprojects were grouped and analysed with two different profilers (Kraken and MetaPhlAn4) to obtain initial species abundance to be used for downstream simulations. The results were projected on the UHGG species representative genomes and used as input for the CAMISIM metagenome simulator, resulting in two reference datasets (refKrak and refMet4, respectively). Each dataset was then analysed with the four compared profilers (Kraken, MetaPhlAn3 mOTUs3 and MetaPhlan4). (**b**) Each compared profiler’s output was then projected to a reference taxonomy, either UHGG or GTDB.

The experimental workflow of our novel methodology benchmark is illustrated below in Fig. 1.

Each one of the profilers analysed here assigns reads and estimates species abundance according to its own catalogue and corresponding taxonomic annotation *native feature space*. This can be different from the reference feature space (i.e. the genomes used in the simulation) because some of the tools’ features may not be represented or may be wrongly assigned. Here, we have explicitly chosen the reference feature space to be independent of those of the studied tools to avoid biases in favour of any tool. More specifically, (i) an initial measure using MetaPhlAn4 and Kraken/UHGG was performed on two public cohorts (see §6.2.1 for more details). (ii) Kraken (alone) was used to choose the most adapted UHGG representative for MetaPhlAn4 (this was not needed for Kraken/UHGG). (iii) Some reads were drawn from those UHGG genomes with CAMISIM. (iv) New profiling was performed on this simulation with the four different tools (Kraken, mOTUs3, MetaPhlAn3 and MetaPhlAn4). (v) All tools’ results were projected onto two common feature spaces: one based on the GTDB taxonomy R207 (*the GTDB feature space*) and the other based on the UHGG catalogue.

Fig. 1(a) illustrates our methodology. In short, we start by computing reference abundance profiles with two different profilers based on real gut metagenomic data from two studies. These abundances along with the set of genomes from UHGG were used to simulate two sets of different metagenomes using CAMISIM, which were next used as input to all compared pipelines for profiling. Fig. 1(b) illustrates the projection procedure, and the potential difficulties involved, notably that a strain/genome can be associated with different species, depending on the feature space used or none. In other words, this means that there is no unique one-to-one correspondence between features of any two spaces, and a feature in one space may potentially correspond to multiple features in another one. Thus, the projection would inevitably introduce errors in estimating species diversity and abundance profiles. We now consider how different steps of this workflow are implemented in our study.

#### Simulating metagenomic data

The source material of the simulation is a typical meta-cohort of real metagenomic data from human faecal samples (*n*=343), comprising two public studies (i.e. PRJEB6070 [12] and PRJEB7774 [11]) including patients with colorectal cancer (*n*=180) and controls (*n*=163). Two different simulations were run using CAMISIM [43] based on the initial species abundance profiles obtained with two different tools: MetaPhlAn4 and Kraken+Bracken/UHGG (i.e. Kraken using the UHGG collection, version 2.0.1). The term feature was retained to designate how species/clades/OTU is described by the different tools.

The two simulated datasets were, respectively, named refMet4 and refKrak. For refMet4, profiles were based upon the internal catalogue features not directly translatable to the UHGG collection, so a representative species in the UHGG was found using Kraken/UHGG (alone) on marker genes for each feature. For refKrak, the number of identified species was much higher; therefore, some filtering steps were added in Bracken post-treatment as suggested by the authors [49]: all species with less than 2000 reads (-t 2000) were discarded as this was the minimal filtering to ensure no sample had more than a thousand species, which is an upper bound for species richness in refMet4. The Kraken tendency of overestimating species richness was already reported in previous studies [53] and was also confirmed in the present work (see the ‘Results’ section). For each dataset, 343 simulated samples composed of 10 million 2×150 bp paired-end reads were generated with CAMISIM v1.3 using the HiSeq profile. In total, 686 (343×2) different samples were simulated. The general characteristics of the simulated data are illustrated in Figs S4 and S5.

#### Abundance quantification and projection

Each simulated dataset was analysed with four different profilers: Kraken+Bracken/GTDB (Kraken version: 2.1.2, Bracken version: 2.8; the catalogue used is the GTDB catalogue in this case), MetaPhlAn3 (version 3.1.0), mOTUs3 (version 3.0.3) and MetaPhlAn4 (version 4.0.6). The abundance estimation of the features in the respective native feature spaces was obtained.

#### Projection to GTDB feature space

MetaPhlAn3, MetaPhlAn4 and mOTUs3 features were projected to GTDB applying Kraken/GTDB (alone) on marker genes associated with their native features. MetaPhlAn4 embarks its own ad hoc projection tool (sgb_to_gtdb_profile.py); however, we chose to use the same methodology for all pipelines. For Kraken, no projection was needed as GTDB is its native feature space.

#### Projection to UHGG feature space

Similarly, all pipeline results were projected onto UHGG feature space with Kraken/UHGG (alone) on marker genes associated with their native features. In the specific case of the projection of the Kraken/GTDB catalogue to UHGG, using only representative genomes was not sufficient; the complete catalogue of marker genes had to be used. When projecting with Kraken/UHGG, all associated features were retained (splitting results equally on all the identified features). The final projected tables can be found in the project git repository (see the ‘Data Summary’ section).

#### Converting abundance tables to the common feature space

**Fig. 2.**
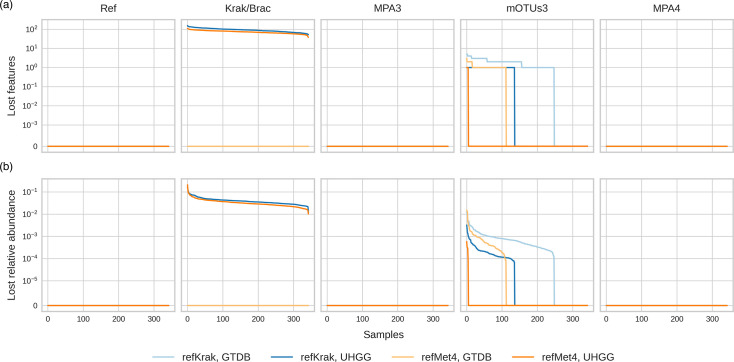
The number of features (**a**) and the relative abundance (**b**) lost when projecting the abundance tables from the native feature space to the two common feature spaces for the simulation source (identified as Ref in the figure) and the four tools. Samples are ordered independently according to the number of lost species and abundance for each of the simulations and common feature space.

When converting the abundance tables from the tools’ native feature space to the common feature spaces, the following procedure was followed:

- Abundances of the native features corresponding to the same feature of the common feature space were summed.
- Abundances of the native features corresponding to several features of the native feature space were split proportionally. The proportions were obtained during the projection (this was necessary only for conversion to UHGG, as this case does not appear from the projection onto the GTDB space).
- The native features without correspondence in the common feature space were omitted, and the abundance tables obtained after conversion were normalized to unity (see Fig. 2 and its discussion in the ‘Results’ section).
- We omitted all the features that could not be identified at the species level.

#### Computing performance metrics

The final metrics were calculated using Python packages NumPy, SciPy, Biopython and Scikit-bio (the code is provided as part of the Supplementary materials). All the figures were created using Matplotlib.

*Sensitivity* and *precision* are defined as

$\text{Sensitivity}=\frac{TP}{TP+FN};\;\text{Precision}=\frac{TP}{TP+FP},$ (1)

where *TP*, *FP* and *FN* are the numbers of true positives (features correctly identified), false positives (features identified but not present in the samples) and false negatives (features not identified, although present in the samples), respectively.

The *false-positive relative abundance (FPRA*) is the sum of the estimated relative abundances of false positives in a sample. Similarly, the *false-negative relative abundance (FNRA)* is the sum of the reference relative abundances of false negatives.

*Bray–Curtis distance* is defined by

$d_{BC}(u,v)=\frac{\sum_{i}\left|u_{i}-v_{i}\right|}{\sum_{i}\left|u_{i}+v_{i}\right|},$ (2)

where *u_i_* and *v_i_* are the true and the estimated abundances of species *i*.

*Weighted UniFrac distance* [54,55] is defined as

$d_{WUF}(u,v)=\frac{\sum_{i=1}^{n}b_{i}\left|u_{i}-v_{i}\right|}{\sum_{i=1}^{n}b_{i}(u_{i}+v_{i})},$ (3)

where *b_i_* is the length of the branch *i* on the phylogenetic tree relating all the features. The phylogenetic trees for calculating UniFrac distances in GTBD and UHGG feature spaces were taken from the respective repositories (https://data.gtdb.ecogenomic.org/releases/release207/207.0/) and (http://ftp.ebi.ac.uk/pub/databases/metagenomics/mgnify_genomes/human-gut/v2.0.1/phylogenies/).

The *Aitchison distance* was calculated as an Euclidean distance between clr-transformed data [56,58]. As the microbial data are highly sparse, the zeros were replaced using a multiplicative replacement strategy [59], as recommended in skbio.stats.composition package (https://scikit.bio/docs/dev/generated/skbio.stats.composition.html#module-skbio.stats.composition).

*Jensen–Shannon divergence* is the pairwise distance between two matrices *p* and *q*. It is defined as

$JSD(p,q)=\sqrt{\frac{D(p∥m)+D(q∥m)}{2}},$ (4)

where *m* = (*P+q*)*/*2, and the Kullback–Leibler divergence is given by

$D(p∥m)=\sum_{i,j}p_{ij}\text{log}\left(\frac{p_{ij}}{q_{ij}}\right),$ (5)

Identifying frequently confused species

*False positives* (FP) are species that have zero abundance in the simulation but have nonzero abundance in the estimation. *False negatives* (FN) are those that are present in the simulation but have zero abundance in the estimation. To determine if any species are systematically confused with one another, we correlated the abundances of false-positive species in the simulation with those of false positives in the estimation across the samples. For any pair of species (one FN and one FP), we considered only the samples where the former has zero abundance in the estimation (i.e. it is an FP), whereas the latter has zero abundance in the simulation (i.e. it is an FP). Indeed, in the samples where both species appear either in the simulation or in the reference, one of them cannot be considered as false positive or false negative.

We required at least 25 samples with identified confusion to calculate the correlations and that the Pearson correlation coefficient exceeds 50% (or 90% for the cases presented in Fig. 7(c). The full list of such confusion pairs, occurring in different simulations, projection spaces and for different tools, can be found in the supplementary data.

### Task orchestration

The computational burden behind the generation and processing of high sequencing depth simulated samples (3Gbp) required an orchestration solution, enabling the distribution of computational tasks over different servers, with the ability to quickly integrate different scientific programs (i.e. the abovementioned state-of-the-art taxonomic profilers and CAMISIM). Different orchestration and workflow solutions were tested (including Nextflow [60] and Celery [61]), and while these solutions have strong qualities, the amount of reworking required for each pipeline and some performance issues made them not suited for our purpose. A *de novo* orchestration solution was developed upon the simple idea of a distributed task queue of containerized tasks, which is now proposed as an open-source solution: scitq, provided in the context of the present work (see the ‘Data summary’ section).

## Results

### Features lost in projection

We encountered several notable challenges in our examination of microbiome profiling tools, each operating with distinct reference catalogues and native feature spaces. The primary objective was to assess the comparative performance of the four profiling tools using simulated metagenomics data. These selected tools represent a subset of a larger pool of available options (see Table S1). We observed that no single common feature space could impartially represent all profiling tools, thereby inevitably introducing biases that could favour some tools over others. Additionally, we noted that the choice of the tool used to generate the simulation could further influence the performance assessment. To ensure a fair and comprehensive evaluation of these tools, we meticulously considered these influential factors. Consequently, we devised an experimental framework encompassing two distinct simulations, each driven by reference abundance data generated by a different tool (refKrak for Kraken and refMet4 for MetaPhlAn4), in conjunction with two common feature spaces, GTDB (release R207) and UHGG representant genomes (version 2.01) [28,52], resulting in a total of four distinct projections (see the ‘Methods’ section). The UHGG genome collection was also used as a genome source for simulation material. While UHGG is specific to the human gut microbiome, the European Bioinformatics Institute (EMBL-EBI) MGnify collection to which it belongs proposes different catalogues for different biome contexts, which gives the opportunity to adapt this method for those other contexts.

We initiated our assessment by examining the impact of projections from each native feature space onto the two common feature spaces. As illustrated in Fig. 2a, b facet *Ref*, all reference features were correctly projected. This observation extends to both versions of MetaPhlAn. However, this was not the case for mOTUs3 and Kraken (Fig. 2a, b facets 2 : 6).

### Richness and Shannon diversity estimation

**Fig. 3.**
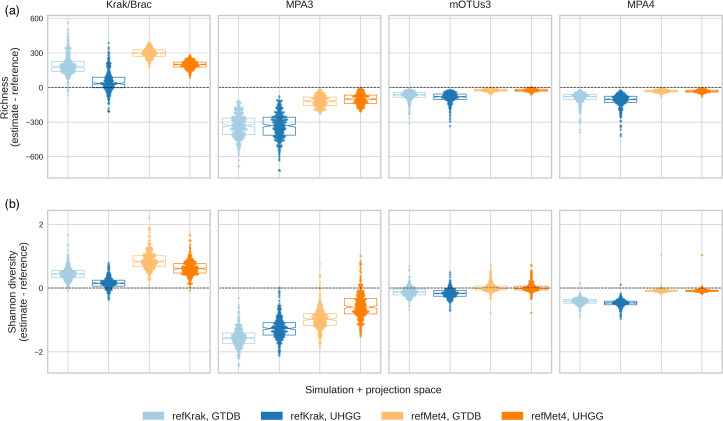
(**a**) Difference of the estimated species richness with the reference richness depicted by boxplots and jittered points. (**b**) Difference of the estimated Shannon diversity with the reference Shannon diversity depicted by boxplots and jittered points.

Richness and Shannon diversity are two widely used metrics in microbiome studies to estimate the complexity of the studied ecosystems. Microbiome diversity is shown to play an important role not only in the health of the ecosystem but also associates with a healthier phenotype of the host [10,62]. The simulation-based approach used here allowed us not only to compare the performance of the four studied profilers in terms of accuracy but also to evaluate the impact of the common feature spaces. Our observations revealed notable discrepancies among the tools. Specifically, the Kraken pipeline consistently exhibited a significant overestimation of both richness and Shannon diversity, while MetaPhlAn3 exhibited underestimations, which is consistent with several previous comparisons (see Table S2). Comparatively, the remaining tools (mOTUs3 and MetaPhlAn4) demonstrated smaller deviations from the ground truth. They performed more effectively in the refMet4 than in the refKrak simulation, potentially due to its heightened complexity (see Fig. 3). mOTUs3 exhibited the highest accuracy.

### Abundance estimation performance

**Fig. 4.**
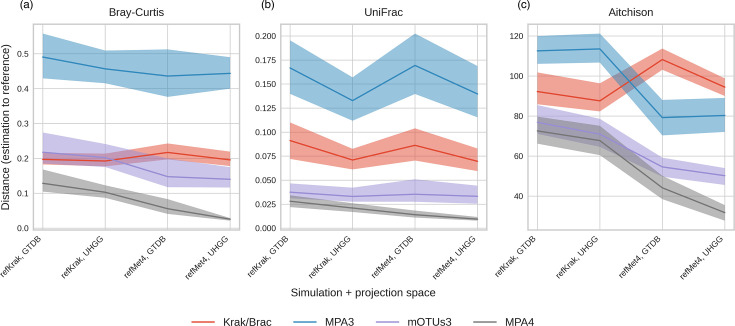
Bray–Curtis (**a**), UniFrac (**b**) and Aitchison (**c**) pairwise distances between the estimated and the reference abundance profiles, displayed as mean ± sd. Colours depict the five different tools.

As microbiome data inherently exhibit a compositional nature, the precise estimation of species proportions remains a pivotal requirement for microbiome profiling tools. We examined the impact of simulations and common feature spaces on the tools’ ability to provide accurate abundance estimation. This assessment was based on two widely used distance metrics, namely, Bray–Curtis and the weighted UniFrac [54,55], which quantify the dissimilarity between the estimated abundance profiles and the ground truth (see Fig. 4).

MetaPhlAn3 displayed the highest level of discrepancy in accurately estimating species proportions, followed by Kraken and mOTUs3, while MetaPhlAn4 performed better. In terms of UniFrac distances, Kraken consistently lagged behind mOTUs3, which approached the performance levels of MetaPhlAn4. Notably, UniFrac distances minimized differences compared with Bray–Curtis distances across simulations and common feature spaces, as seen in Fig. S1. This suggests that incorrect species identifications often involved phylogenetically closely related species, especially for mOTUs3.

For the most accurate tools (mOTUs3 and MetaPhlAn4), a principal coordinate analysis (PCoA) exploring the differences in beta diversity between the reference and the estimation was also conducted (see Fig. S2).

### Impact of simulation in species discovery and abundance estimation

**Fig. 5.**
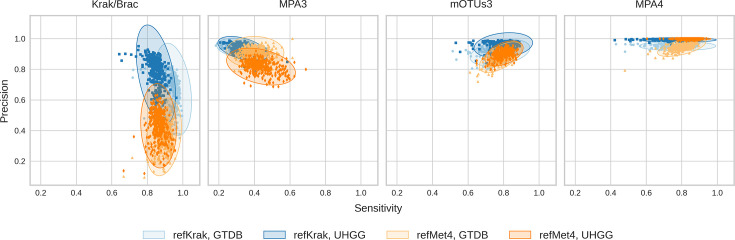
Sensitivity and precision evaluation of the five tools and the impact of the simulations and common feature spaces. Each point corresponds to a sample, in the simulation defined by the colour scheme in the legend. Error ellipses indicate sd of the sample distribution.

After the overall estimation of relative abundance, we evaluated the impact of the simulation and the common feature spaces on the ability of the studied profilers to correctly discover the abundance and presence of the microbial species. Sensitivity and precision, depicted in Fig. 5, respectively, measure the tools’ ability to identify truly present species and avoid false positives, e.g. species erroneously detected by a pipeline (a boxplot representation of the same analysis is provided in Fig. S6). Kraken emerged as one of the most sensitive tools, indicating a lower rate of false negatives, although its specificity (e.g. the number of false positives) was notably impacted by the simulation, particularly in the case of refMet4. However, the influence of the common feature space was less pronounced. In contrast, MetaPhlAn3 exhibited lower sensitivity, although its precision remained high. mOTUs3 demonstrated lower precision than MetaPhlAn4 but struck a balance between false positives and false negatives. MetaPhlAn4, on the other hand, leaned towards precision over sensitivity with overall very good performance.

**Fig. 6.**
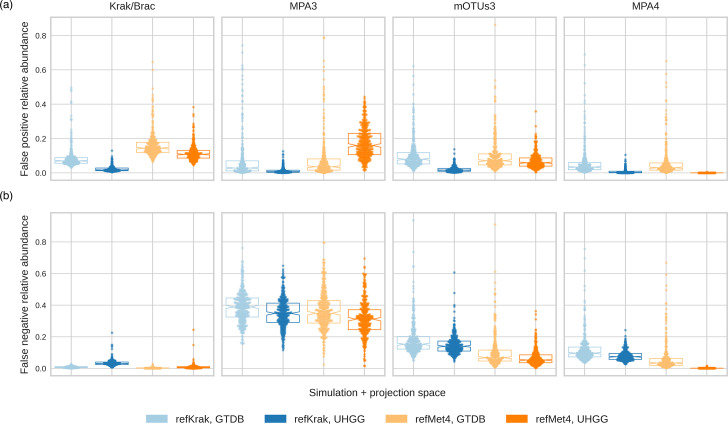
(a) False-positive relative abundance, computed as the cumulative abundance of the false-positive matches between the estimated abundance and the ground truth. This indicates the proportion of relative abundance that is attributed to species that were not present in the simulation. (**b**) False-negative relative abundance computed from the ground truth. This indicates the proportion of relative abundance of species present in the simulation but either non-detected or confused with another species. Colours indicate the different simulations and common feature spaces across the five profiling tools.

Moreover, we evaluated the impact of the simulation on the compared tools, on their ability to estimate species abundance. For this, we computed the cumulative relative abundance of the false-positive matches (FPRA). We observed a significant number of outlier samples with FPRA as high as 80%, but only when using the GTDB feature space (see Fig. 6).

**Fig. 7.**
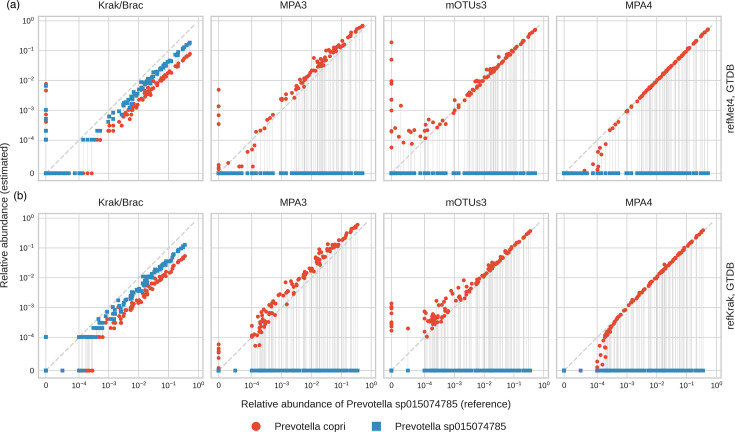

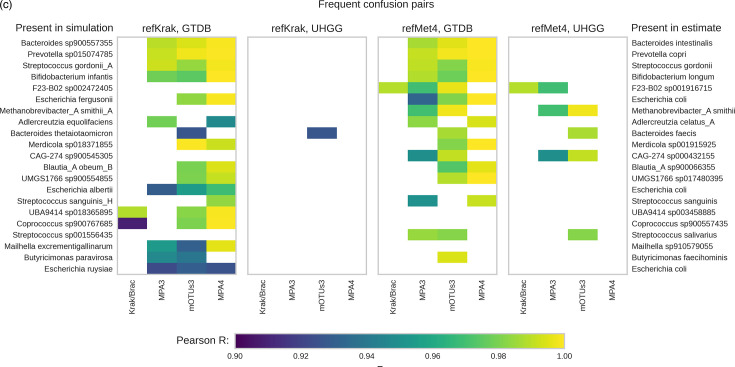
(a) and (b) Estimated abundance of two *Prevotella copri* and *Prevotella* sp*.015074785* vs. the abundance of the latter in the simulated data. When working in GTDB feature space, *P.* sp*.015074785* (rather abundant in the simulation) is systematically mistaken for *P. copri* (absent in the simulation.). The only exception is Krak/Brac pipeline, which splits the abundance of *P.* sp*.015074785* between the two species. (Vertical lines indicate the abundances of the two species in corresponding samples.) (c) The most frequently confused pairs of species by simulation, feature space and the pipeline (see the main text for methodology).

This high error rate is primarily due to a small number of closely related species that were wrongly matched, which is consistent with our earlier observations regarding the UniFrac vs. Bray–Curtis distances. An example of such confusion is illustrated in Fig. 7, where the estimated abundance of *Prevotella* sp*.015074785* and *Prevotella copri* are compared with the abundance of *Prevotella* sp*.015074785* in the simulation. *P. copri* was not present in this simulation, as it did not exist in the UHGG collection (see the ‘Methods’ section). On the other hand, *P.* sp*.015074785* was misidentified by both MetaPhlAns and mOTUs3 as *P. copri*. Kraken found both species in correlated proportions. However, none of them was equal to their reference abundances.

We found additional mistaken species by correlating the abundances of false positives and false negatives (i.e. abundances of species present in a simulation but not in the estimation with those of the species present in estimations but not in the ground truth – see the ‘Methods’ section for more details). The frequently recurring pairs of such species with high correlation are shown in Fig. 7c.

### Community structure

A frequently used method for representing community structure involves conducting a PCoA of the pairwise distance matrix. Fig. S2 illustrates the first two components of this decomposition for MetaPhlAn4 and mOTUs3. However, since the initial principal components only capture a portion of the information embedded in the pairwise distance matrix, we opted for a direct comparison of these matrices. This comparison involved calculating pairwise distance matrices for the reference simulation profiles and the estimations provided by each tool.

**Fig. 8.**
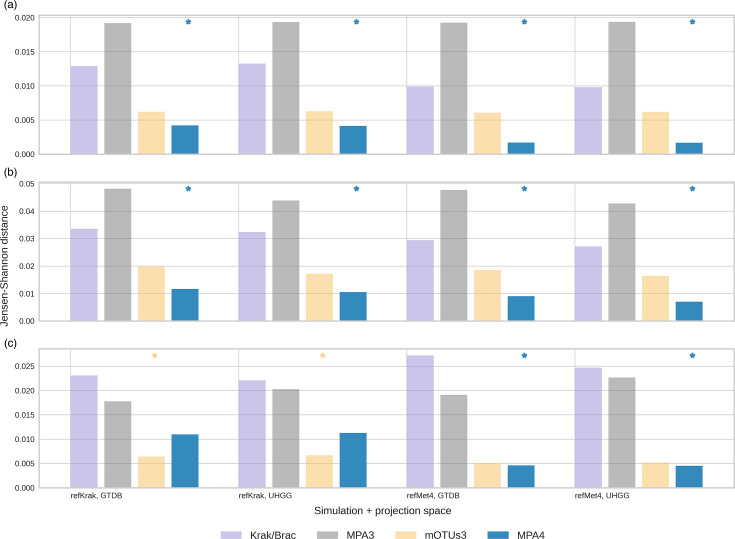
Similarity between pairwise distance matrices for the simulated data and the estimations. (**a**) Bray–Curtis pairwise distances, (**b**) weighted UniFrac pairwise distances, (**c**) Aitchison pairwise distances. Best tools are annotated with a ‘*’.

We employed the Jensen–Shannon distance to measure dissimilarity between the pairwise distance matrices for ground truth and the estimates. When using Bray–Curtis or UniFrac distances, the top-performing tool was MetaPhlAn4 with mOTUs3, Kraken and MetaPhlAn3 following in that order as illustrated in Fig. 8. The situation is different when using Aitchison distance: MetaPhlAn3 outperforms Kraken for all the simulations and spaces, whereas mOTUs3 is better than MetaPhlAn4 when refKrak simulation is used. It is necessary to point out that the greatest contributions to Aitchison distance come from the clades that are present in one sample but absent in another one (and must probably be imputed by a small value close to the detection threshold – see the ‘Methods’ section). Thus, the MetaPhlAn3 limited feature space has the effect of bringing the samples closer, while in the rich feature space of kraken, false positives/negatives have the effect of significantly increasing distances between samples. The mOTUs3 prevailing over MetaPhlAn4 in refKrak simulation reflects the near parity between these tools observed throughout our analyses.

### Summary tables and tool ranking

Table 1 summarizes the median values (median across the samples) of the abundance and features lost in projecting onto the common feature space, as well as the abundance and Shannon diversity of the projected data. Table 2 summarizes the median values of the results presented above. Based on different metrics, the tools were ranked as follows:

1. *Richness estimate*. Kraken consistently overestimated species richness, whereas MetaPhlAn3 systematically underestimated it. MetaPhlAn4 tended to underestimate richness. mOTUs3 was the best in this category.
2. *Shannon diversity*. Kraken overestimated the Shannon diversity, while MetaPhlAn3 underestimated it. MetaPhlAn4 also underestimated it, while mOTUs3 proved the most accurate.
3. *Bray–Curtis distance*. MetaPhlAn3 was furthest from the ground truth, followed by Kraken and mOTUs3, with MetaPhlAn4 being closest to the ground truth.
4. *UniFrac distance*. MetaPhlAn3 was furthest from the ground truth, followed by Kraken, mOTUs3, and MetaPhlAn4.
5. *Taxonomic sensitivity*. Kraken had the best sensitivity. MetaPhlAn4 and mOTUs3 performed similarly. MetaPhlAn3 had the lowest sensitivity (it generated a higher number of false negatives).
6. *Taxonomic precision*. MetaPhlAn4 was the most precise tools, followed by MetaPhlan3 (due to its small catalogue), by mOTUs3 and then by Kraken (producing a higher number of false positives).
7. *FPRA*. Kraken had the greatest abundance of false positives, followed by mOTUs3 and MetaPhlAn3. Both MetaPhlAns performed similarly. FPRA was significantly lower in the UHGG feature space.
8. *False-negative relative abundance (FNRA)*. Kraken had overall the lowest occurrence of false negative, MetaPhlAn4 came after, with a significantly higher occurrence (three times at last), and then mOTUs, not very far behind, and MetaPhlAn3 showed the highest occurrence.

**Table 1.** Median values (across the samples) of lost abundance, lost features, richness and Shannon diversity

Tool	Simulation	Common space	Lost abundance	Lost clades	Richness	Shannon
Ref	refMet4	GTDB	0.0000	0	214	5.5574
Ref	refMet4	UHGG	0.0000	0	215	5.5574
Ref	refKrak	GTDB	0.0000	0	489	6.6404
Ref	refKrak	UHGG	0.0000	0	512	6.6848
mOTUs3	refMet4	GTDB	0.0000	0	191	5.5497
mOTUs3	refMet4	UHGG	0.0000	0	190	5.5513
mOTUs3	refKrak	GTDB	0.0004	1	420	6.4917
mOTUs3	refKrak	UHGG	0.0000	0	428	6.4972
MPA4	refMet4	GTDB	0.0014	4	176	5.4500
MPA4	refMet4	UHGG	0.0290	5	175	5.4420
MPA4	refKrak	GTDB	0.0025	12	392	6.2038
MPA4	refKrak	UHGG	0.0345	12	392	6.1695
MPA3	refMet4	GTDB	0.0000	0	91	4.5695
MPA3	refMet4	UHGG	0.0685	8	105	4.9009
MPA3	refKrak	GTDB	0.0000	0	159	5.0266
MPA3	refKrak	UHGG	0.0859	13	174	5.3352
Krak/Brac	refMet4	GTDB	0.0000	0	521	6.4017
Krak/Brac	refMet4	UHGG	0.0304	72	419	6.1869
Krak/Brac	refKrak	GTDB	0.0000	0	692	7.0674
Krak/Brac	refKrak	UHGG	0.0377	92	574	6.8477

**Table 2.** Median values (across the samples) of major metrics (error in estimated richness and Shannon diversity, Bray–Curtis and UnifFrac distances between the estimations and the reference, sensitivity, precision, FPRA and FNRA)

Tool	Simulation	Space	Richness diff.	Shannon diff.	Bray–Curtis	UniFrac	Sensitivity	Precision	FPRA	FNRA
Krak/Brac	refMet4	GTDB	299	0.8331	0.2171	0.0863	0.8966	0.3659	0.1436	0.0018
Krak/Brac	refMet4	UHGG	200	0.6129	0.1957	0.0696	0.8642	0.4425	0.1075	0.0060
Krak/Brac	refKrak	GTDB	179	0.4414	0.1972	0.0912	0.9517	0.6966	0.0698	0.0076
Krak/Brac	refKrak	UHGG	34	0.1501	0.1925	0.0710	0.8515	0.7955	0.0166	0.0304
MPA3	refMet4	GTDB	−118	−0.9837	0.4361	0.1694	0.3962	0.9172	0.0357	0.3489
MPA3	refMet4	UHGG	−107	−0.6763	0.4704	0.1629	0.4000	0.8193	0.1688	0.3544
MPA3	refKrak	GTDB	−331	−1.5697	0.4907	0.1668	0.3021	0.9455	0.0281	0.3887
MPA3	refKrak	UHGG	−341	−1.3598	0.4865	0.1578	0.3127	0.9387	0.0040	0.3871
mOTUs3	refMet4	GTDB	−23	−0.0070	0.1476	0.0353	0.7853	0.8868	0.0710	0.0678
mOTUs3	refMet4	UHGG	−24	−0.0079	0.1400	0.0332	0.8056	0.9091	0.0587	0.0529
mOTUs3	refKrak	GTDB	−64	−0.1239	0.2177	0.0375	0.7930	0.9179	0.0793	0.1525
mOTUs3	refKrak	UHGG	−80	−0.1661	0.2023	0.0331	0.8115	0.9650	0.0157	0.1395
MPA4	refMet4	GTDB	−35	−0.0994	0.0463	0.0117	0.8083	0.9732	0.0176	0.0275
MPA4	refMet4	UHGG	−37	−0.1355	0.0505	0.0207	0.8212	1.0000	0.0000	0.0319
MPA4	refKrak	GTDB	−91	−0.4335	0.1196	0.0260	0.7923	0.9756	0.0194	0.0880
MPA4	refKrak	UHGG	−115	−0.5176	0.1300	0.0322	0.7749	0.9955	0.0001	0.1050

#### Best profilers on our radar

**Fig. 9.**
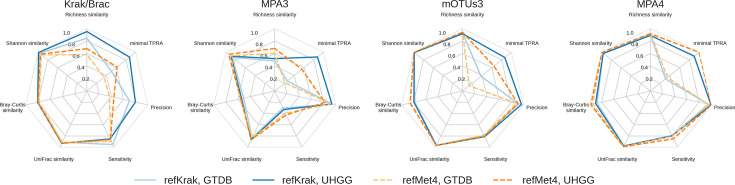
Spider web illustrations of the comparison of tool performances across different metrics, simulations and projection spaces.

Figs 9 and S3 compare the performance of the profiling tools studied across different metrics, simulations and projections spaces. To facilitate the representation, we calculated Bray–Curtis similarity for richness and Shannon diversity estimates (i.e. we present 1 − |*u − v*|*/*(*u+v*), where *u,v* are the true and the estimated richness/Shannon diversity). Bray–Curtis and UniFrac distances to the ground truth were also transformed to similarities as 1−*d_BC_*, 1 − *d_WUF_*. Likewise, instead of FPRA, we took the minimal abundance of false positives across the samples, *TPRA*=1 − *FPRA.* (For all other metrics, we present the median value.) Finally, no transformation was necessary for sensitivity and precision, which naturally change between 0 and 1, with higher values corresponding to the best results. The results are also presented in Tables S3–S10.

## Discussion

The benchmarking approach we designed and explored in this article revolves around the concept of common feature spaces and the impact of simulation on various microbiome profiling tools. We implemented different standardized metrics to compare their ability to estimate the abundance and prevalence of microbiome features.

The loss of a certain number of features and their corresponding abundance in the projections remains relatively low across all tools (see Fig. 2). This observation holds true when comparing the median number of lost features to the median total number of features (richness), notably in the case of GTDB feature space.

The absence of a one-to-one correspondence between different feature spaces is prone to generate discrepancies, such as false positives and false negatives, with the extent of these discrepancies contingent on the proximity of the native and projection feature spaces. To mitigate the impact of such disparities on the evaluation of profiling tools, we devised an experimental framework comprising different simulations and common feature spaces. The performance of the tools was markedly influenced by the simulations. However, it is evident that Kraken+Bracken and MetaPhlAn3 consistently lagged in performance, irrespective of the simulation or projection space chosen. Conversely, the two remaining tools (mOTUs3 and MetaPhlAn4) demonstrated significantly superior performance, with variations across simulations.

We conducted a further assessment to quantify the extent of these disparities by computing distances between the estimated relative abundance and the ground truth, utilizing both Bray–Curtis and phylogenetically sensitive weighted UniFrac distances (Figs 4 and S1). Notably, the observation that UniFrac distances exhibit greater resilience to the impact of various simulations compared with Bray–Curtis distances suggests that mismatches primarily arise from phylogenetically closely related features. These discrepancies can be attributed to differences in the categorization of individual genomes or strains into species, as illustrated in Fig. 1(b). Such mismatches assume reduced significance when analyses are conducted at higher taxonomic levels, such as genus or family, as is commonly practised in benchmarking studies.

Specifically, we illustrated that the elevated levels of FPRA detected in certain samples could be wholly attributed to the confusion between phylogenetically closely related features. In instances where one feature exhibited high abundance in the simulation, this phenomenon became particularly evident (see Fig. 7).

The Kraken+Bracken pipeline consistently exhibited a tendency to identify an excessive number of false positives. This resulted in an overestimation of diversity metrics (richness and Shannon), elevated distances (Bray–Curtis and UniFrac) from the reference abundance profile and a lower precision score. Some filtration options are recommended for Kraken usage, using either a minimizer score (Kraken FAQ) or confidence score [44], so Kraken could have been optimized more, likely with a cost on sensitivity. However, our purpose here is to analyse a naive use of the tool with default options, so we did not filter results. Nevertheless, this pipeline compensated for these drawbacks with its high sensitivity. Kraken proved to be exceptionally valuable in facilitating cross-referencing between different feature spaces (see the ‘Methods’ section), highlighting its versatility in utilizing various catalogues, including both GTDB and UHGG, as an advantageous trait.

Although still widely used, MetaPhlAn3 [44,63, 64] (along with its previous versions [36,37, 45,47, 65]; see Table S2) did not withstand the competition with more recent tools. Its primary handicap stemmed from its highly constricted native feature space, resulting in a substantial number of false negatives, thereby impairing all performance metrics.

Among the two most effective tools studied here (mOTUs3 and MetaPhlAn4), only mOTUs3 was not given the advantage of a simulation using its own measurements. This was primarily due to resource constraints and secondly because its simulation results would have been quite like MetaPhlAn4’s (both are not specialized marker-gene-based pipelines, and MetaPhlAn4 is more recent). Nevertheless, mOTUs3 displayed little variation across the simulations and the projection spaces (see Fig. 4). Importantly, mOTUs3 struck a balance between the numbers of false negatives and false positives, reflecting a good trade-off between sensitivity and precision. In terms of Bray–Curtis distance from the ground truth, mOTUs3 was similar to Kraken but clearly outperformed this pipeline when using the phylogenetically sensitive UniFrac distance. Additionally, the error in estimating species richness or Shannon diversity remained relatively insensitive to the simulation and the projection.

Overall, MetaPhlAn4 exhibited strong performance, excelling in specificity with minimal false positives. However, this specificity came at the cost of sensitivity, as it demonstrated relatively higher numbers of false negatives with notable variability across the simulations. MetaPhlAn4 did not appear to significantly benefit from being used for the simulation and was disadvantaged in terms of richness and Shannon diversity estimation when the simulation was based on Kraken. Furthermore, projecting MetaPhlAn4 onto the UHGG feature space resulted in a disadvantage, both in diversity estimates and in proximity to the ground truth.

The performance of the profiling tools exhibited sensitivity to the simulation details. This sensitivity can be attributed, in part, to the specific features chosen for representation in the simulations, such as cases where certain tools could not distinguish between two species of *Prevotella* or had differing annotations in the simulation and tool catalogues. Additionally, feature size in the simulations varied, with refKrak containing more than twice as many features as refMet4. While this variability did not impact our conclusions about Kraken and MetaPhlAn3, it did influence the performance of the remaining tools. Overall, mOTUs3 displayed the most consistent performance across the simulations, while MetaPhlAn4 was notably affected by the feature-rich refKrak simulation, which may include numerous species that are not present in its catalogue. It is worth noting that the high number of false positives generated by Kraken potentially makes the refKrak simulated dataset the least realistic. MetaPhlAn4 exhibited improved performance when the simulation was based on its own measurements (see Fig. 4).

When comparing the performance of the tools, the choice of a common feature space had a notably greater impact than the initial tool selected for the simulation. MetaPhlAn4 exhibited a significant advantage when projected onto the UHGG feature space compared with the GTDB projection (see Fig. 5). This relative advantage was more pronounced than that observed for any other pipeline, with Kraken, for instance, experiencing only marginal improvements from using its native space. Our interpretation is that the choice of a common feature space fundamentally alters the nature of the comparison, whereas the initial choice of the profiling tool is more of a technical detail. The GTDB projection is better suited for assessing tools’ performance in a general bacterial detection context, while the UHGG projection better represents their performance in the specific context of human gut microbiome detection. Therefore, MetaPhlAn, being partially specialized for this environment, benefits more significantly from this projection advantage than Kraken.

In this study, we aimed to explore a fundamental conceptual question within the field, necessitating substantial computational resources and reliance on extensive public catalogues. Given the inherent complexity of the question, we intentionally limited our focus to the human gut microbiome context. However, we acknowledge this limitation as a constraint of our study and advocate for applying this approach to evaluate additional catalogues and tools across diverse microbiome settings. We have explored in detail the false-positive defects of the different tools but could not do the same for false negative: we think an unrealistic simulation with a gradual increase in the relative abundance of different species would be more adapted to this task. Because of the use of UHGG as a source of genome, eukaryotes and viruses are not included in this simulation, and the performance of the different tools is not assessed for either.

## supplementary material

10.1099/mgen.0.001330 Uncited Supplementary Material 1.

## Abbreviations

BC: biological context
Bracken: Bayesian Reestimation of Abundance with KrakEN
EBI: European Bioinformatics Institute
EMBL: European Molecular Biology Laboratory
FN: false negatives
FNRA: false-negative relative abundance
FP: false positives
FPRA: false-positive relative abundance
GTDB: Genome Taxonomy Database
NCBI: National Center for Biotechnlogy Information
NCBI SRA: NCBI's Sequence Read Archive
OTUs: operational taxonomic units
PCoA: principal coordinate analysis
UHGG: Unified Human Gastrointestinal Genome
WMS: whole metagenomic sequencing
