# Supplementary material for: Impact of simulation and reference catalogues on the evaluation of taxonomic profiling pipelines
Source: Microb Genom. 2025 Jan 13;11(1):001330. doi: 10.1099/mgen.0.001330 (PMC11728698; doi:10.1099/mgen.0.001330)
Supplement: Uncited Supplementary Material 1. [file mgen-11-01330-s001.pdf]

Impact of simulation and reference catalogues on the evaluation of taxonomic profiling pipelines

Supplementary Material

Author names

Vadim Puller[1] (0000-0002-3900-8283), Florian Plaza Oñate[1] (0000-0001-6036-0989), Edi Prifti[2,3,*] (0000-0001-8861-1305) and Raynald de Lahondès[1,*](0009-0000-2862-9589)

Affiliation(s)

[1]GMT science, 75 route de Lyons-La-Foret, Rouen, F-76000, France

[2]IRD, Sorbonne Université, Unité de Modélisation Mathématique et Informatique des Systèmes Complexes, UMMISCO, 32 avenue Henri Varagnat, Bondy, F-93143, France

[3]Sorbonne Université, INSERM, Nutrition et Obesities; systemic approaches, NutriOmique, AP-HP, Hôpital Pitié-Salpêtrière, 91 boulevard de l'Hôpital, Paris, F-75013, France

1 Supplementary figures

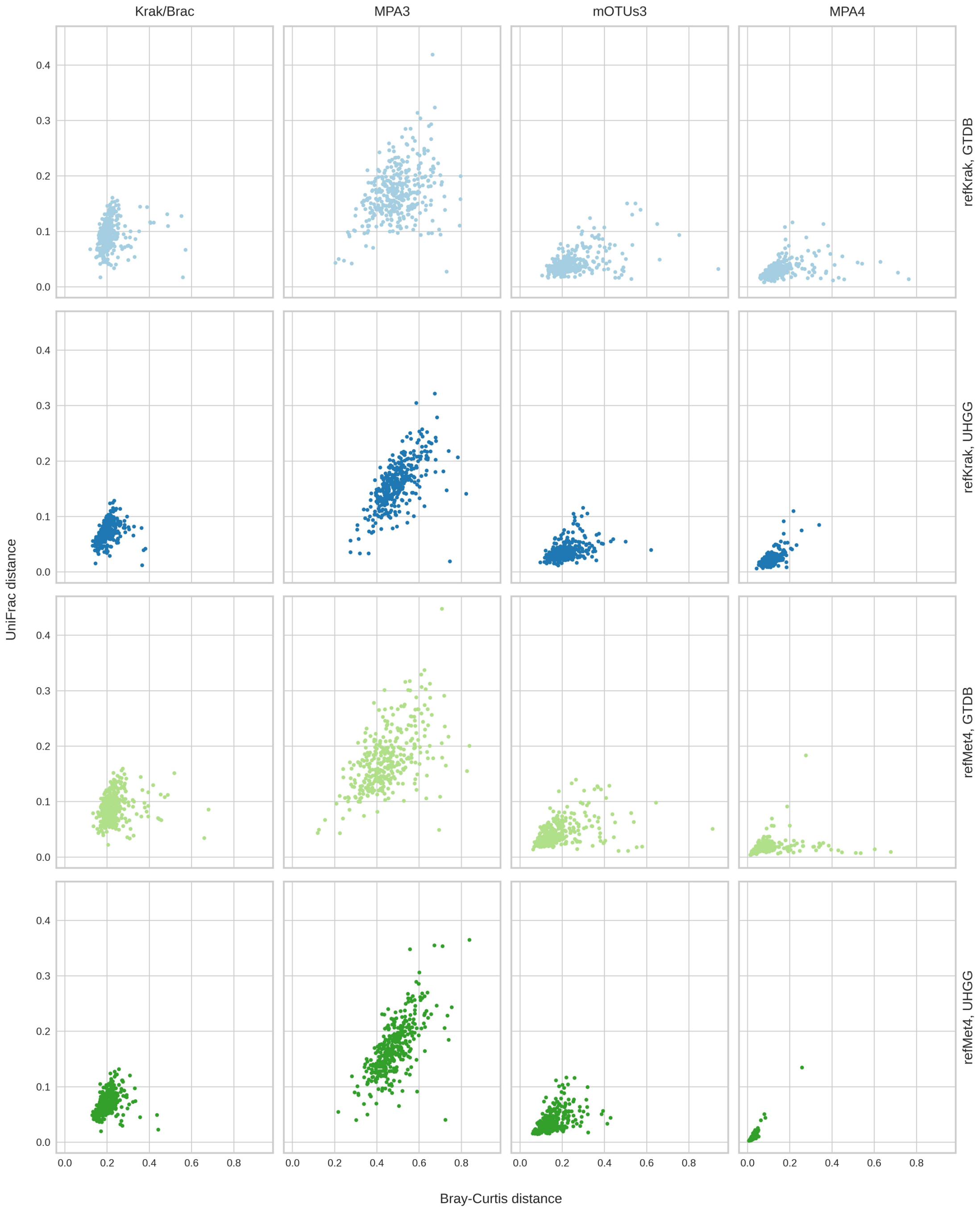

Figure S1 Scatterplot of weighted UniFrac distance from estimation to reference vs. the Bray- Curtis distance. Each point corresponds to a sample.

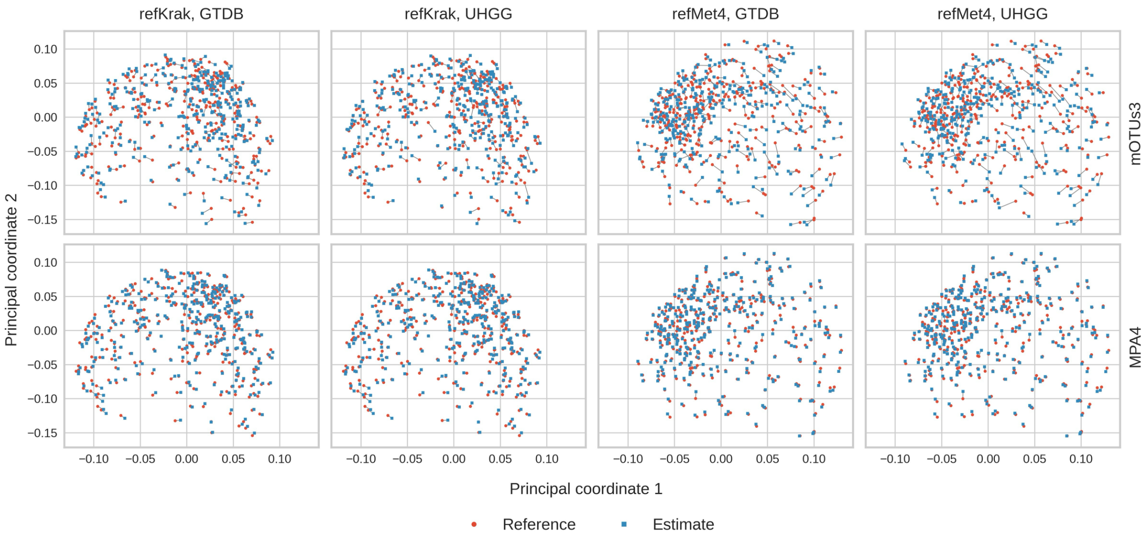

Figure S2 Principal Coordinates decomposition of pairwise distance matrices (Bray-Curtis distance). Thin lines, connecting the points for the same sample in the ground truth and the estimate, are intended as a guide for the eye.

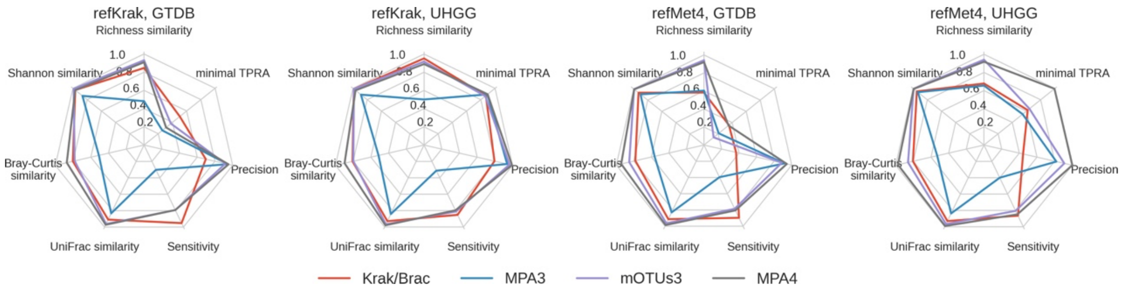

Figure S3 Comparison of tool performances across different metrics, simulations, and projection spaces.

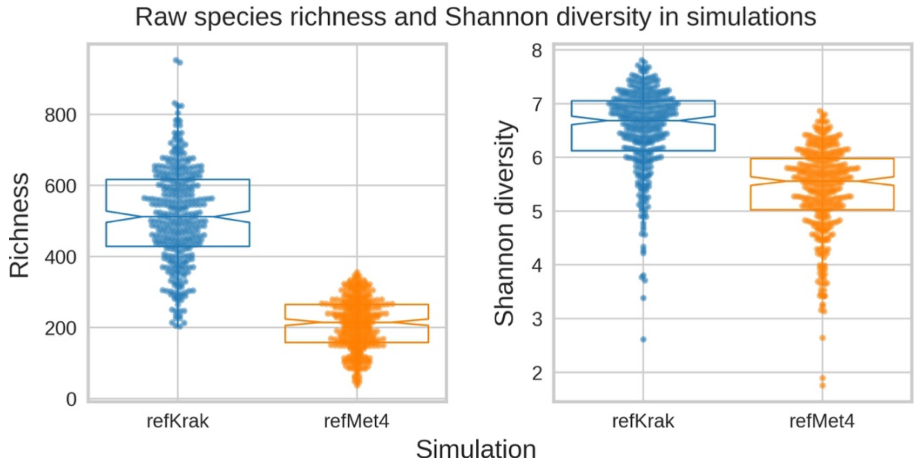

Figure S4 Raw species richness and Shannon diversity in the simulated data.

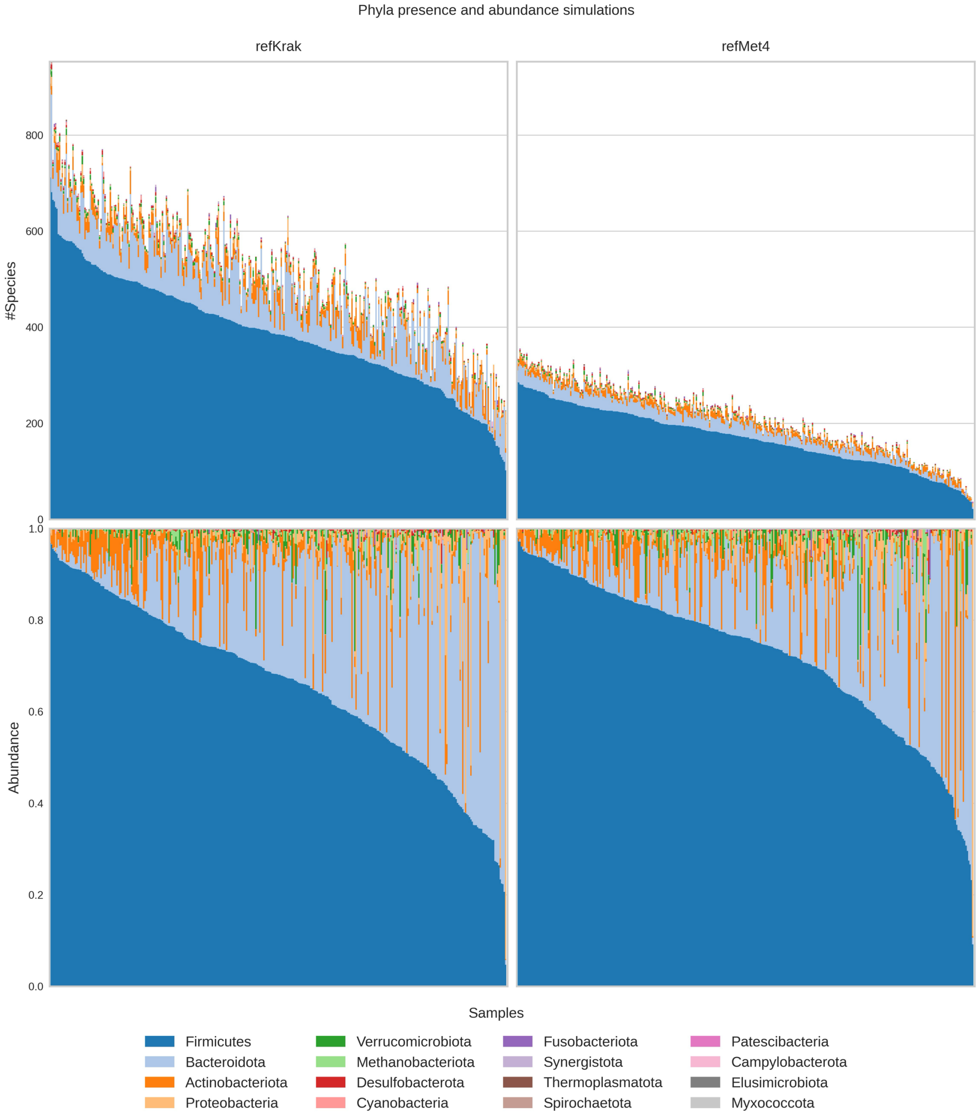

Figure S5 Presence and abundance of bacterial phyla in the simulated data.

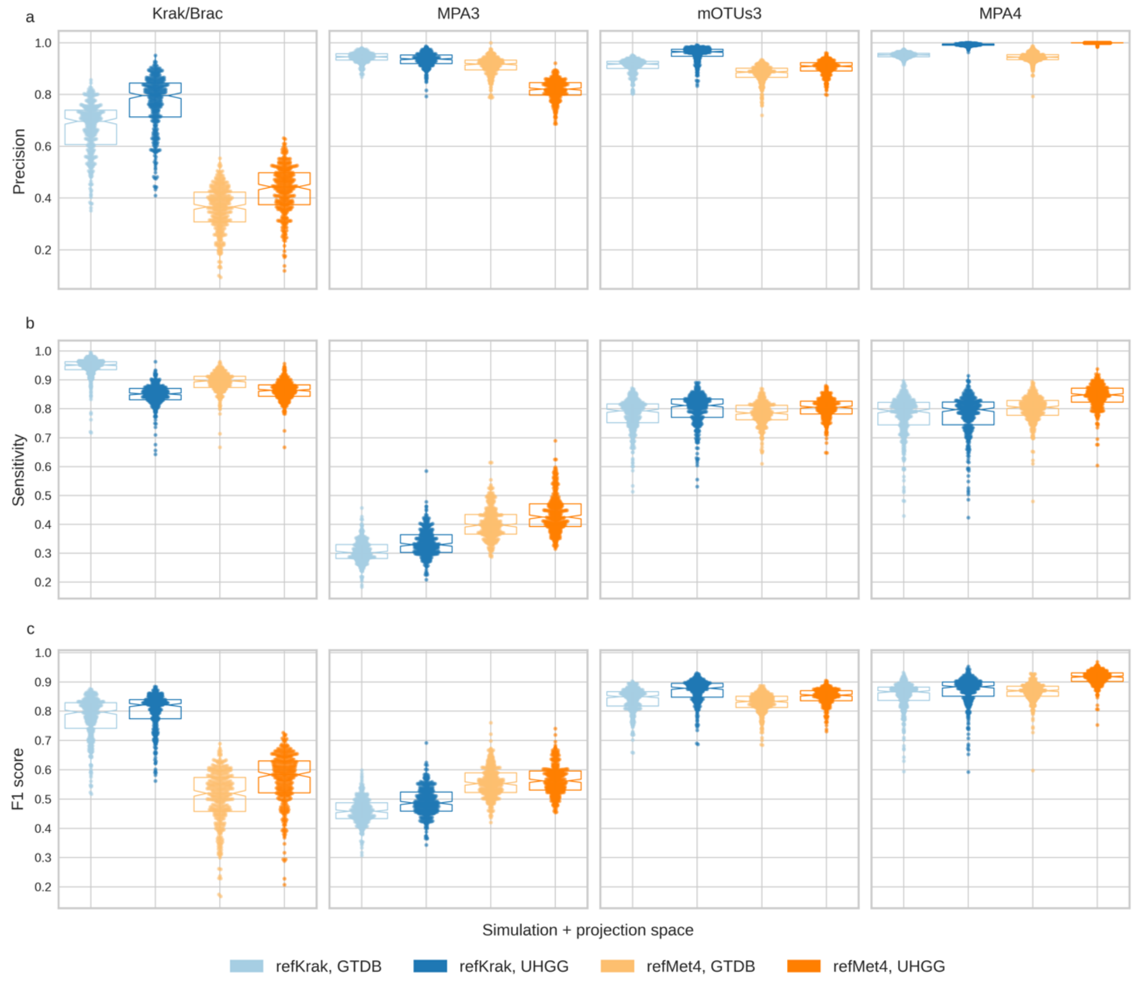

Figure S6 Precision, Sensitivity and F1 score of the different pipelines according to the simulation and projection.

2 Overview of the most popular metagenomic tools

The table below summarizes some of the most popular metagenomic tools, as reviewed in (1–11). For clarity the tools that provides profiling from WGS data only through use of plugins (such as QIIME (12,13) and NGLess (14)) have been excluded. Tools relying on contig assembly (such as metaBAT (15,16), CONCOCT (17) or MaxBin (18,19)) were also excluded. We briefly describe the columns of these tables:

2.1 Stand alone

Some of these tools require pre-processing, i.e., they function as a part of a pipeline with other tools: e.g., Bracken is a profiling tool used with Kraken output. Moreover, several tools are not available for download, but function as web-servers, where the users may download their data for processing.

2.2 Function

Here we refer to the tools that only determine presence of species as *classification* tools, whereas those that determine the abundance profile/prevalence as *profiling tools*.

2.3 Method

Marker genes and *whole genome* refers to the tools mapping reads to marker genes or whole genomes, with the implication that marker genes usually constitute a tool-specific catalogue (even if borrowed from a public database), whereas whole genome tools are generally capable of working with a user-specified genome catalogue. For Bracken and MEGAN, which function in pipeline with Kraken 2 and DIAMOND respectively, this field is not specified.

The remaining columns designate the year when the first version of tool was published and cite the relevant publications.

Table S1 Most popular metagenomic tools (as reviewed in (1–6,20–22))

Tool	Stand alone	Function	Method	Year	Reference
Kraken	yes	classification	whole genome	2014	(23,24)
Bracken	use with Kraken	profiling		2017	(25,26)
mOTUs	yes	profiling	marker genes	2013	(11,27)
MetaPhlAn	yes	profiling	marker genes	2012	(28–30)
MetaPhlAn4	yes	profiling	marker genes	2022	(31,32)
MetaPhyler	yes	profiling	marker genes	2011	(33,34)
TIPP	yes	profiling	marker genes	2014	(35,36)
FOCUS	yes	profiling	whole genome	2014	(37)
DIAMOND	yes	classification	whole genome	2015	(38)
MEGAN6	use with DIAMOND or BLAST	profiling		2007	(39–45)
MetaPalette	yes	profiling	whole genome	2016	(46)
GOTTCHA	yes	profiling	marker genes ?	2015	(47)
MICROBA	proprietary software	profiling	whole genome	2021	(4)
METEOR	Yes	profiling	non-redondant	2010	(48,49)
MOCAT2	yes	profiling	whole genome	2012	(50,51)
Centrifuge	yes	classification	whole genome	2016	(52)
CLARK	yes	classification	whole genome	2015	(53,54)
Kaiju	yes	classification	whole genome	2016	(55)
NBC++	Web-server	classification	whole genome	2010	(56,57)
PhymmBL	yes	classification	whole genome	2009	(58,59)
Ganon	yes	classification	whole genome	2020	(60)
DUDes	yes	classification	whole genome	2016	(61)
Metacache	yes	classification	whole genome	2017	(62)
LMAT	yes	classification	whole genome	2013	(63–65)
LCA*	classifies asembled contigs	classification	whole genome	2016	(66)
MetaWatt	bin assembled contigs	classification	contigs	2012	(67)
taxator-tk	use with BLAST or assembled contigs	classification	whole genome	2015	(68)
CCMetagen	yes	classification	whole genome	2020	(69,70)
specI	Web-server	classification	marker genes	2013	(71)

3 State-of-the-art benchmarking studies

In **Table S2** we provide a short summary of the benchmarking studies involving metagenomic profilers (1–7,9–11,20–22,72).

3.1 Diversity of profilers and versions

Table S1 presents only part of the tools analysed in the cited benchmarking studies. The number of tools compared in a given study varies from two (particularly where it mainly concerns introducing a new tool (11)) to several dozen (21,73). Various versions of Kraken (and Bracken), mOTUs and MetaPhlAn appear in most studies and can be viewed as the most popular representatives of the most recognized algorithms. However, the published studies, although recent, consider older versions of

these tools: mOTUs2 and MetaPhlAn3 or even lower. This is less of a problem for Kraken, which remains unchanged since the emergence of Kraken2 and Bracken.

3.2 Correspondence between feature spaces

Establishing correspondence between the feature space in which simulation is performed and the native feature spaces of the studied profilers is crucial for using most of the canonical performance metrics. Indeed, even simple metrics such as the species richness, is highly dependent on the catalogue used (*i.e.* the same number of genomes can be grouped into different number of species or be grouped differently; see **Figure 9b** in the main text). To our knowledge the influence of the feature space used for comparison had not been previously studied. Previous state-of-the-art studies focused exclusively on the tools using the NCBI taxonomy, or performed taxonomic comparison at higher taxonomic levels - family or genus (where the discrepancy is less visible, and the performance is consequently better than when focusing at the species level, see, e.g., (11)). This noticeably put at disadvantage mOTUs, using its own original taxonomy (1,21), and, to a lesser extent MetaPhlAn, mostly based on the NCBI taxonomy. (This shortcoming is alleviated only partially by using UniFrac distance, and almost never alongside the related, but phylogenetically insensitive, Bray-Curtis distance.)

3.3 The impact of the reference databases

A Recent study (4) used the GTDB database as a standardized feature catalogue for all tools that were compared (4). However, although this permitted the comparison between the classification/profiling algorithms, it deprived some of them from any advantage that they might have had using their own unique catalogues. The influence of the database choice has been recently the focus of interest in (8,10). However, these studies were limited to varying the size of the Kraken2 database, and always focused on the same feature space (GTDB or NCBI).

3.4 Simulated samples and mock communities

CAMI and CAMI2 (5,6,73,74) are the two benchmarking datasets that were explicitly designed for comparing metagenomic pipelines and used in numerous studies (2,5,8,10,11,21). Other studies have used their own simulations (4,7,20) or sometimes combination of own simulations and CAMI datasets (2,8,11). Finally, there are studies that used the data obtained from synthetic communities (3,9,22). The number of samples in these studies could vary from a few samples, e.g., (1,7) to several hundred (4,5,21). The number of species in simulated samples was typically a few hundred, whereas in synthetic samples only a few dozen or less.

In terms of the effort made to make simulated samples to represent the real- world data, those based on CAMISIM simulator are probably the most advanced. However, no specific study until now had used CAMISIM for generating its own samples (with the obvious exceptions of the

studies carried within the context of CAMI challenges (5,21)). Finally, the studies vary greatly in terms of the type of the microbiome addressed: ocean, plant, human gut, mouse gut or simply generic microbiome based on the reference genome database.

In the table below we have sometimes used improper references to the tools such as Kraken2 or mOTUs2 to denote the major version that was used in the corresponding study.

Table S 2 Previous benchmarking studies

Study	Method of comparison	Test data	Number of samples	Type of microbiome	Reference taxonomy	Number of tools	Tools studied	Reference
Lindgreen (2016)	different taxonomic ranks	own simulations	6	unspecified	NCBI	14	Kraken, MetaPhlAn, mOTU, other	(20)
Sczyrba (2017)	different taxonomic ranks	CAMI challenge	700	unspecified	NCBI	10	Kraken (binning only), MetaPhlAn2, mOTUs, other	(21)
McIntyre (2017)	different taxonomic ranks	synthetic and simulations	6	human mouth, human gut, other	NCBI	11	Kraken, MetaPhlAn2, other	(22)
Ye (2019)	standartized database, different taxonomic ranks	own simulations	10	unspecified	NCBI	18	Kraken2+Bracken, MetaPhlAn2, mOTUs2, other	(1)
Milanese (2019)	different taxonomic ranks	own simulations, CAMI challenge	10	human gut	NCBI	11	Kraken+Bracken, MetaPhlAn2, mOTUs2, other	(11)
Seppey (2020)	imposing NCBI taxonomy	own simulations, CAMI1	8	unspecified	NCBI	8	Kraken2+Bracken, MetaPhlAn2, other	(2)
Amos (2020)	limited number of known species	synthetic communities	2	human gut	NCBI	5	Kraken+Bracken, MetaPhlAn2, other	(3)
Miossec (2020)	different taxonomic ranks	own simulations	8	gut, ocean	NCBI	8	Kraken, MetaPhlAn2, other	(7)
Parks (2021)	standartized database	own simulations	140	unspecified, gut	GTDB, NCBI	10	Kraken2+Bracken, MetaPhlAn2, mOTUs2	(4)
Meyer (2022)	different taxonomic ranks	CAMI II	113	human microbiome, mouse gut	NCBI	20	Kraken2+Bracken, MetaPhlAn2, mOTUs2, other	(5,6)
Portik (2022)	limited number of known species, different taxonomic leveles	synthetic communities	10	unspecified	NCBI	10	Kraken2, MetaPhlAn3, mOTUs2, other	(9)
Wright (2023)	same taxonomy	CAMI, other	164	mouse gut, marine, other	NCBI, GTDB	2	Kraken2+Bracken, MetaPhlAn3	(8)
Xu (2023)	different taxonomic ranks	CAMI, biological samples	10	mouse gut	NCBI	9	Kraken2+Bracken, MetaPhlAn3, other	(10)

4 Ranking tools

We ranked the performance of the tools according to different metrics by per- forming pairwise one-sided Wilcoxon sign tests. The results are presented in the tables S3-S10.

Table S3 Ranking of the tools according to the error in estimating species richness.

Simulation	Common space	Best	2nd best	3rd best	Last
refKrak	GTDB	mOTUs3	MPA4	Krak/Brac	MPA3
refKrak	UHGG	Krak/Brac	mOTUs3	MPA4	MPA3
refMet4	GTDB	mOTUs3	MPA4	MPA3	Krak/Brac
refMet4	UHGG	mOTUs3	MPA4	MPA3	Krak/Brac

Table S4 Ranking of the tools according to the error in estimating Shannon diversity.

Simulation	Common space	Best	2nd best	3rd best	Last
refKrak	GTDB	mOTUs3	Krak/Brac	MPA4	MPA3
refKrak	UHGG	Krak/Brac	mOTUs3	MPA4	MPA3
refMet4	GTDB	mOTUs3	MPA4	Krak/Brac	MPA3
refMet4	UHGG	mOTUs3	MPA4	Krak/Brac	MPA3

Table S5 Ranking of the tools according to the Bray-Curtis distance from the ground truth.

Simulation	Common space	Best	2nd best	3rd best	Last
refKrak	GTDB	MPA4	Krak/Brac	mOTUs3	MPA3
refKrak	UHGG	MPA4	Krak/Brac	mOTUs3	MPA3
refMet4	GTDB	MPA4	mOTUs3	Krak/Brac	MPA3
refMet4	UHGG	MPA4	mOTUs3	Krak/Brac	MPA3

Table S6 Ranking of the tools according to the weighted UniFrac distance from the ground truth.

Simulation	Common space	Best	2nd best	3rd best	Last
refKrak	GTDB	MPA4	mOTUs3	Krak/Brac	MPA3
refKrak	UHGG	mOTUs3	MPA4	Krak/Brac	MPA3
refMet4	GTDB	MPA4	mOTUs3	Krak/Brac	MPA3
refMet4	UHGG	MPA4	mOTUs3	Krak/Brac	MPA3

Table S7 Ranking of the tools according to their sensitivity.

Simulation	Common space	Best	2nd best	3rd best	Last
refKrak	GTDB	Krak/Brac	mOTUs3	MPA4	MPA3
refKrak	UHGG	Krak/Brac	mOTUs3	MPA4	MPA3
refMet4	GTDB	Krak/Brac	MPA4	mOTUs3	MPA3
refMet4	UHGG	Krak/Brac	MPA4	mOTUs3	MPA3

Table S 8 Ranking of the tools according to their precision.

Simulation	Common space	Best	2nd best	3rd best	Last
refKrak	GTDB	MPA4	MPA3	mOTUs3	Krak/Brac
refKrak	UHGG	MPA4	mOTUs3	MPA3	Krak/Brac
refMet4	GTDB	MPA4	MPA3	mOTUs3	Krak/Brac
refMet4	UHGG	MPA4	mOTUs3	MPA3	Krak/Brac

Table S 9 Ranking of the tools according to the false positive relative abundance.

Simulation	Common space	Best	2nd best	3rd best	Last
refKrak	GTDB	MPA4	MPA3	Krak/Brac	mOTUs3
refKrak	UHGG	MPA4	MPA3	Krak/Brac	mOTUs3
refMet4	GTDB	MPA4	MPA3	mOTUs3	Krak/Brac
refMet4	UHGG	MPA4	mOTUs3	Krak/Brac	MPA3

Table S 10 Ranking of the tools according to the false negative relative abundance.

Simulation	Common space	Best	2nd best	3rd best	Last
refKrak	GTDB	Krak/Brac	MPA4	mOTUs3	MPA3
refKrak	UHGG	Krak/Brac	MPA4	mOTUs3	MPA3
refMet4	GTDB	Krak/Brac	MPA4	mOTUs3	MPA3
refMet4	UHGG	Krak/Brac	MPA4	mOTUs3	MPA3

References

1. Simon HY, Siddle KJ, Park DJ, Sabeti PC. Benchmarking metagenomics tools for taxonomic classification. Cell. 2019;178(4):779-94.
2. Seppey M, Manni M, Zdobnov EM. LEMMI: a continuous benchmarking platform for metagenomics classifiers. Genome Res. 2020;30(8):1208-16.
3. Amos GC, Logan A, Anwar S, Fritzsche M, Mate R, Bleazard T, et al. Developing standards for the microbiome field. Microbiome. 2020;8:1-13.
4. Parks DH, Rigato F, Vera-Wolf P, Krause L, Hugenholtz P, Tyson GW, et al. Evaluation of the microba community profiler for taxonomic profiling of metagenomic datasets from the human gut microbiome. Front Microbiol. 2021;12:643682.
5. Meyer F, Fritz A, Deng ZL, Koslicki D, Lesker TR, Gurevich A, et al. Critical assessment of metagenome interpretation: the second round of challenges. Nat Methods. 2022;19(4):429-40.
6. McHardy AC, Meyer F. CAMI II: identifying best practices and issues for metagenomics software. Vol. 19, NATURE METHODS. NATURE PORTFOLIO HEIDELBERGER PLATZ 3, BERLIN, 14197, GERMANY; 2022. p. 412-3.
7. Miossec MJ, Valenzuela SL, Pérez-Losada M, Johnson WE, Crandall KA, Castro-Nallar E. Evaluation of computational methods for human microbiome analysis using simulated data. PeerJ. 2020;8:e9688.
8. Wright RJ, Comeau AM, Langille MG. From defaults to databases: parameter and database choice dramatically impact the performance of metagenomic taxonomic classification tools. Microb Genomics. 2023;9(3).
9. Portik DM, Brown CT, Pierce-Ward NT. Evaluation of taxonomic classification and profiling methods for long-read shotgun metagenomic sequencing datasets. BMC Bioinformatics. 2022;23(1):541.
10. Xu R, Rajeev S, Salvador LC. The selection of software and database for metagenomics sequence analysis impacts the outcome of microbial profiling and pathogen detection. Plos One. 2023;18(4):e0284031.
11. Milanese A, Mende DR, Paoli L, Salazar G, Ruscheweyh HJ, Cuenca M, et al. Microbial abundance, activity and population genomic profiling with mOTUs2. Nat Commun. 2019;10(1):1-11.
12. Bolyen E, Rideout JR, Dillon MR, Bokulich NA, Abnet CC, Al-Ghalith GA, et al. Reproducible, interactive, scalable and extensible microbiome data science using QIIME 2. Nat Biotechnol. 2019;37(8):852-7.
13. Caporaso JG, Kuczynski J, Stombaugh J, Bittinger K, Bushman FD, Costello EK, et al. QIIME allows analysis of high-throughput community sequencing data. Nat Methods. 2010;7(5):335-6.
14. Coelho LP, Alves R, Monteiro P, Huerta-Cepas J, Freitas AT, Bork P. NG-meta-profiler: fast processing of metagenomes using NGLess, a domain-specific language. Microbiome. 2019;7(1):1-10.
15. Kang DD, Froula J, Egan R, Wang Z. MetaBAT, an efficient tool for accurately reconstructing single genomes from complex microbial communities. PeerJ. 2015;3:e1165.
16. Kang DD, Li F, Kirton E, Thomas A, Egan R, An H, et al. MetaBAT 2: an adaptive binning algorithm for robust and efficient genome reconstruction from metagenome assemblies. PeerJ. 2019;7:e7359.
17. Alneberg J, Bjarnason BS, De Bruijn I, Schirmer M, Quick J, Ijaz UZ, et al. Binning metagenomic contigs by coverage and composition. Nat Methods. 2014;11(11):1144-6.
18. Wu YW, Tang YH, Tringe SG, Simmons BA, Singer SW. MaxBin: an automated binning method to recover individual genomes from metagenomes using an expectation-maximization algorithm. Microbiome. 2014;2:1-18.
19. Wu YW, Simmons BA, Singer SW. MaxBin 2.0: an automated binning algorithm to recover genomes from multiple metagenomic datasets. Bioinformatics. 2016;32(4):605-7.
20. Lindgreen S, Adair KL, Gardner PP. An evaluation of the accuracy and speed of metagenome analysis tools. Sci Rep. 2016;6(1):19233.
21. Sczyrba A, Hofmann P, Belmann P, Koslicki D, Janssen S, Dröge J, et al. Critical assessment of metagenome interpretation—a benchmark of metagenomics software. Nat Methods. 2017;14(11):1063-71.
22. McIntyre AB, Ounit R, Afshinnekoo E, Prill RJ, Hénaff E, Alexander N, et al. Comprehensive benchmarking and ensemble approaches for metagenomic classifiers. Genome Biol. 2017;18(1):1-19.
23. Wood DE, Salzberg SL. Kraken: ultrafast metagenomic sequence classification using exact alignments. Genome Biol. 2014;15(3):1-12.
24. Wood DE, Lu J, Langmead B. Improved metagenomic analysis with Kraken 2. Genome Biol. 2019;20(1):1-13.

25. Lu J, Breitwieser FP, Thielen P, Salzberg SL. Bracken: estimating species abundance in metagenomics data. PeerJ Comput Sci. 2017;3:e104.
26. Lu J, Rincon N, Wood DE, Breitwieser FP, Pockrandt C, Langmead B, et al. Metagenome analysis using the Kraken software suite. Nat Protoc. 2022;1-25.
27. Ruscheweyh HJ, Milanese A, Paoli L, Sintsova A, Mende DR, Zeller G, et al. mOTUs: Profiling Taxonomic Composition, Transcriptional Activity and Strain Populations of Microbial Communities. Curr Protoc. 2021;1(8):e218.
28. Segata N, Waldron L, Ballarini A, Narasimhan V, Jousson O, Huttenhower C. Metagenomic microbial community profiling using unique clade-specific marker genes. Nat Methods. 2012;9(8):811-4.
29. Truong DT, Franzosa EA, Tickle TL, Scholz M, Weingart G, Pasolli E, et al. MetaPhlAn2 for enhanced metagenomic taxonomic profiling. Nat Methods. 2015;12(10):902-3.
30. Beghini F, McIver LJ, Blanco-Míguez A, Dubois L, Asnicar F, Maharjan S, et al. Integrating taxonomic, functional, and strain-level profiling of diverse microbial communities with bioBakery 3. Elife. 2021;10:e65088.
31. Blanco-Miguez A, Beghini F, Cumbo F, McIver LJ, Thompson KN, Zolfo M, et al. Extending and improving metagenomic taxonomic profiling with uncharacterized species with MetaPhlAn 4. bioRxiv. 2022;
32. Truong DT, Tett A, Pasolli E, Huttenhower C, Segata N. Microbial strain-level population structure and genetic diversity from metagenomes. Genome Res. 2017;27(4):626-38.
33. Liu B, Gibbons T, Ghodsi M, Pop M. MetaPhyler: Taxonomic profiling for metagenomic sequences. In: 2010 IEEE international conference on bioinformatics and biomedicine (BIBM). IEEE; 2010. p. 95-100.
34. Liu B, Gibbons T, Ghodsi M, Treangen T, Pop M. Accurate and fast estimation of taxonomic profiles from metagenomic shotgun sequences. Genome Biol. 2011;12:1-27.
35. Nguyen N phuong, Mirarab S, Liu B, Pop M, Warnow T. TIPP: taxonomic identification and phylogenetic profiling. Bioinformatics. 2014;30(24):3548-55.
36. Shah N, Molloy EK, Pop M, Warnow T. TIPP2: metagenomic taxonomic profiling using phylogenetic markers. Bioinformatics. 2021;37(13):1839-45.
37. Silva GGZ, Cuevas DA, Dutilh BE, Edwards RA. FOCUS: an alignment-free model to identify organisms in metagenomes using non-negative least squares. PeerJ. 2014;2:e425.
38. Buchfink B, Xie C, Huson DH. Fast and sensitive protein alignment using DIAMOND. Nat Methods. 2015;12(1):59-60.
39. Gautam A, Felderhoff H, Bağci C, Huson DH. Using AnnoTree to get more assignments, faster, in DIAMOND+MEGAN microbiome analysis. Msystems. 2022;7(1):e01408-21.
40. Gautam A, Zeng W, Huson DH. MeganServer: facilitating interactive access to metagenomic data on a server. Bioinformatics. 2023;39(3):btad105.
41. Huson DH, Auch AF, Qi J, Schuster SC. MEGAN analysis of metagenomic data. Genome Res. 2007;17(3):377-86.
42. Huson DH, Mitra S, Ruscheweyh HJ, Weber N, Schuster SC. Integrative analysis of environmental sequences using MEGAN4. Genome Res. 2011;21(9):1552-60.
43. Huson DH, Beier S, Flade I, Górska A, El-Hadidi M, Mitra S, et al. MEGAN community edition-interactive exploration and analysis of large-scale microbiome sequencing data. PLoS Comput Biol. 2016;12(6):e1004957.
44. Huson DH, Albrecht B, Bağcı C, Bessarab I, Gorska A, Jolic D, et al. MEGAN-LR: new algorithms allow accurate binning and easy interactive exploration of metagenomic long reads and contigs. Biol Direct. 2018;13(1):1-17.
45. Arumugam K, Bağcı C, Bessarab I, Beier S, Buchfink B, Górska A, et al. Annotated bacterial chromosomes from frame-shift-corrected long-read metagenomic data. Microbiome. 2019;7(1):1-13.
46. Koslicki D, Falush D. Metapalette: ak-mer painting approach for metagenomic taxonomic profiling and quantification of novel strain variation. MSystems. 2016;1(3):e00020-16.
47. Freitas TAK, Li PE, Scholz MB, Chain PS. Accurate read-based metagenome characterization using a hierarchical suite of unique signatures. Nucleic Acids Res. 2015;43(10):e69-e69.
48. Pons N, Batto J, Kennedy S, Almeida M, Boumezbeur F, Moumen B, et al. METEOR, a platform for quantitative metagenomic profiling of complex ecosystems [Internet]. 2010. Disponible sur: http://www.jobim2010.fr/sites/default/files/presentations/27Pons.pdf
49. Gauthier F, Pons N. Meteor (Metagenomic Explorator), a software for profiling metagenomic data at gene level [Internet]. 2021. Disponible sur: https://forgemia.inra.fr/metagenopolis/meteor

50. Kultima JR, Sunagawa S, Li J, Chen W, Chen H, Mende DR, et al. MOCAT: A Metagenomics Assembly and Gene Prediction Toolkit. PLOS ONE. oct 2012;7(10):1-6.
51. Kultima JR, Coelho LP, Forslund K, Huerta-Cepas J, Li SS, Driessen M, et al. MOCAT2: a metagenomic assembly, annotation and profiling framework. Bioinformatics. 2016;32(16):2520-3.
52. Kim D, Song L, Breitwieser FP, Salzberg SL. Centrifuge: rapid and sensitive classification of metagenomic sequences. Genome Res. 2016;26(12):1721-9.
53. Ounit R, Wanamaker S, Close TJ, Lonardi S. CLARK: fast and accurate classification of metagenomic and genomic sequences using discriminative k-mers. BMC Genomics. 2015;16(1):1-13.
54. Ounit R, Lonardi S. Higher classification sensitivity of short metagenomic reads with CLARK-S. Bioinformatics. 2016;32(24):3823-5.
55. Menzel P, Ng KL, Krogh A. Fast and sensitive taxonomic classification for metagenomics with Kaiju. Nat Commun. 2016;7(1):11257.
56. Rosen GL, Reichenberger ER, Rosenfeld AM. NBC: the Naive Bayes Classification tool webserver for taxonomic classification of metagenomic reads. Bioinformatics. 2011;27(1):127-9.
57. Rosen GL, Sokhansanj BA, Polikar R, Bruns MA, Russell J, Garbarine E, et al. Signal processing for metagenomics: extracting information from the soup. Curr Genomics. 2009;10(7):493-510.
58. Brady A, Salzberg SL. Phymm and PhymmBL: metagenomic phylogenetic classification with interpolated Markov models. Nat Methods. 2009;6(9):673-6.
59. Brady A, Salzberg S. PhymmBL expanded: confidence scores, custom databases, parallelization and more. Nat Methods. 2011;8(5):367-367.
60. Piro VC, Dadi TH, Seiler E, Reinert K, Renard BY. ganon: precise metagenomics classification against large and up-to-date sets of reference sequences. Bioinformatics. 2020;36(Supplement_1):i12-20.
61. Piro VC, Lindner MS, Renard BY. DUDes: a top-down taxonomic profiler for metagenomics. Bioinformatics. 2016;32(15):2272-80.
62. Müller A, Hundt C, Hildebrandt A, Hankeln T, Schmidt B. MetaCache: context-aware classification of metagenomic reads using minhashing. Bioinformatics. 2017;33(23):3740-8.
63. Ames SK, Hysom DA, Gardner SN, Lloyd GS, Gokhale MB, Allen JE. Scalable metagenomic taxonomy classification using a reference genome database. Bioinformatics. 2013;29(18):2253-60.
64. Ames S, Allen JE, Hysom DA, Lloyd GS, Gokhale MB. Design and optimization of a metagenomics analysis workflow for NVRAM. In: 2014 IEEE international parallel & distributed processing symposium workshops. IEEE; 2014. p. 556-65.
65. Van Essen B, Hsieh H, Ames S, Gokhale M. DI-MMAP: A high performance memory-map runtime for data-intensive applications. In: 2012 SC Companion: High Performance Computing, Networking Storage and Analysis. IEEE; 2012. p. 731-5.
66. Hanson NW, Konwar KM, Hallam SJ. LCA*: an entropy-based measure for taxonomic assignment within assembled metagenomes. Bioinformatics. 2016;32(23):3535-42.
67. Strous M, Kraft B, Bisdorf R, Tegetmeyer HE. The binning of metagenomic contigs for microbial physiology of mixed cultures. Front Microbiol. 2012;3:410.
68. Dröge J, Gregor I, McHardy AC. Taxator-tk: precise taxonomic assignment of metagenomes by fast approximation of evolutionary neighborhoods. Bioinformatics. 2015;31(6):817-24.
69. Marcelino VR, Clausen PT, Buchmann JP, Wille M, Iredell JR, Meyer W, et al. CCMetagen: comprehensive and accurate identification of eukaryotes and prokaryotes in metagenomic data. Genome Biol. 2020;21(1):1-15.
70. Clausen PT, Aarestrup FM, Lund O. Rapid and precise alignment of raw reads against redundant databases with KMA. BMC Bioinformatics. 2018;19:1-8.
71. Mende DR, Sunagawa S, Zeller G, Bork P. Accurate and universal delineation of prokaryotic species. Nat Methods. 2013;10(9):881-4.
72. Almeida A, Mitchell AL, Boland M, Forster SC, Gloor GB, Tarkowska A, et al. A new genomic blueprint of the human gut microbiota. Nature. 2019;568(7753):499-504.
73. Meyer F, Lesker TR, Koslicki D, Fritz A, Gurevich A, Darling AE, et al. Tutorial: assessing metagenomics software with the CAMI benchmarking toolkit. Nat Protoc. 2021;16(4):1785-801.
74. Fritz A, Hofmann P, Majda S, Dahms E, Dröge J, Fiedler J, et al. CAMISIM: simulating metagenomes and microbial communities. Microbiome. 2019;7(1):1-12.
